# Direct Catalytic Fuel Cell Device Coupled to Chemometric Methods to Detect Organic Compounds of Pharmaceutical and Biomedical Interest

**DOI:** 10.3390/s20133615

**Published:** 2020-06-27

**Authors:** Mauro Tomassetti, Federico Marini, Riccardo Angeloni, Mauro Castrucci, Luigi Campanella, Corrado Di Natale

**Affiliations:** 1 Department of Chemistry, University of Rome “La Sapienza”, P.le A. Moro 5, 00185 Rome, Italy; federico.marini@uniroma1.it (F.M.); r.angeloni_08@libero.it (R.A.); mauro.castrucci@libero.it (M.C.); luigi.campanella@uniroma1.it (L.C.); 2 Department of Electronic Engineering, University of Rome “Tor Vergata”, Via Politecnico 1, 00133 Rome, Italy; dinatale@uniroma2.it

**Keywords:** qualitative and quantitative analyses, fuel cell and chemometrics, pharmaceutical and biomedical compounds

## Abstract

Making use of a small direct methanol fuel cell device (DMFC), used as an analytical sensor, chemometric methods, organic compounds very different from one another, can be determined qualitatively and quantitatively. In this research, the following seven different organic compounds of pharmaceutical and biomedical interest, having in common only one –OH group, were considered: chloramphenicol, imipenem, methanol, ethanol, propanol, atropine and cortisone. From a quantitative point of view, the traditional approach, involving the building of individual calibration curves, which allow the quantitative determination of the corresponding organic compounds, even if with different sensitivities, was followed. For the qualitative analysis of each compound, this approach has been much more innovative. In fact, by processing the data from each of the individual response curves, obtained through the fuel cell, using chemometric methods, it is possible to directly identify and recognize each of the seven organic compounds. Since the study is a proof of concept to show the potential of this innovative methodological approach, based on the combination of direct methanol fuel cell with advanced chemometric tools, at this stage, concentration ranges that may not be the ones found in some real situations were investigated. The three methods adopted are all explorative methods with very limited computation costs, which have different characteristics and, therefore, may provide complementary information on the analyzed data. Indeed, while PCA (principal components analysis) provides the most parsimonious summary of the variability observed in the current response matrix, the analysis of the current response behavior was performed by the “slicing” method, in order to transform the current response profiles into numerical matrices, while PARAFAC (Parallel Factor Analysis) allows to obtain a finer deconvolution of the exponential curves. On the other hand, the multiblock nature of “ComDim” (Common Components and Specific Weight Analysis) has been the basis to relate the variability observed in the current response behavior with the parameters of the linear calibrations.

## 1. Introduction

In the past, several researches have been performed by some authors, with the aim of using different fuel-cell devices for energetic [1,2,3,4,5,6,7,8,9,10,11,12,13,14,15,16,17] and analytical [18,19,20,21,22,23,24] purposes. These researches have effectively shown the possibility to perform some analytical applications but only by means of particular and complex types of fuel cells not useful for common application to real samples. However, our research group recently performed new researches of analytical-quantitative types by using a simple and suitable direct methanol fuel cell (DMFC) [25,26,27]. In some of the works already published [27,28], we have also observed that the cells (DMFC) developed, used or marketed essentially for analytical purposes in the quantitative determination of methanol or ethanol are able to respond, even if with different sensitivities and different response times, to organic compounds other than the simplest aliphatic alcohols, provided that, in their molecule, there is a –OH group having (at least partly) alcoholic properties. This circumstance has prompted us to investigate an idea that we had been trying to develop for some time using other types of sensors; our research team, in fact, has developed many other classic electrochemical sensors and biosensors in the past years, which have generally proven very useful for the quantitative analyses of different compounds, while they have not been so successful in qualitative analyses, both because this analytical aspect has generally been poorly investigated and because some practical difficulties often arise using classic sensors or biosensors and, finally, because modern mathematical methods, such as chemometric ones, have not been applied to this problem. Recently, however, while using the DMFC device for quantitative analytical purposes, we realized that the use of the fuel cell, to the study of qualitative analytical problems like the one addressed in the present manuscript, can have many advantages, especially regarding the reproducibility of the measurements. Just consider, for instance, the fact that, in our case, the sensor must not be immersed in the solution to be tested, an operation that can often impair reproducibility, for example, due to small variations in the hydrostatic pressure on the sensor head. In fact, in the case of the fuel cell, it is the sample that must be placed inside the cell, and this avoids immersion, an operation that is, on the other hand, necessary in the case of other electrochemical sensors. We have therefore taken up the idea of carrying out the qualitative analyses of different organic samples, using a simple fuel cell for the quantitative analysis of ethanol or methanol and applying some of the modern chemometric methods that have proven more suitable for the purposes of qualitative analyses we have proposed; therefore, in the present study, compounds of pharmaceutical and biomedical interest very different from one another, practically having in common only one –OH group, can be determined also qualitatively (as well as, of course, quantitatively), making use of the same small direct methanol-(ethanol) fuel cell (DMFC) employed as an analytical sensor [25].

To this purpose, the following seven organic compounds were considered: chloramphenicol, imipenem, methanol, ethanol, propanol, atropine and cortisone—that is, two antibiotics, three alcohols and two other compounds very important in the biomedical field. From a quantitative point of view, the traditional approach was followed, i.e., building the respective calibration curve for each compound. For the qualitative analysis of single compounds, this approach has been more innovative. In fact, by processing the data from each of the individual response curves obtained through the fuel cell for each of the compounds considered using chemometric methods, it was possible to directly identify and recognize each of the seven studied organic compounds.

## 2. Materials and Methods

### 2.1. Materials and Apparatus

Five of the seven organic compounds (i.e., methanol, ethanol, propanol, atropine and cortisone) were of analytical grade, purchased from Sigma-Aldrich (St. Louis, MO, USA). Two analyzed antibiotics, both injectable pharmaceutical formulations containing chloramphenicol or imipenem, was purchased at a hospital drugstore. We would like to stress that Imipenem can be purchased (as an injectable solution, with a well-known title) exclusively in hospital drugstores, not in common pharmacies nor from the “usual” companies that sell organic substances. Therefore, we purchased both antibiotics, as injectable products with accurately known titles of active substances, from a hospital drugstore in Rome, which granted them to us only for research purposes. On the other hand, we are well-aware that Chloramphenicol can also be purchased from Sigma-Aldrich (Milan, Italy), and in fact, in our studies, we alternatively used both the “commercial” one and that provided by the drugstore hospital, without finding any significant difference in the response of the fuel cell (the difference was lower than the standard deviation reported in Table 1).

All the solutions that have been necessary to build the calibration curves or for recording the response curves were obtained by dissolving directly into the proper measured volume of distilled and deionized water (final conductivity 0.01–0.02 µS) the weighed quantity of each compound.

A DMFC, H-TEC Model F111, fuel cell (50 × 50 × 40 mm and weighing 100 g) obtained from Fuel Cell Store (College Station, TX, USA) was used. The cell was made in Plexiglas©, while the electrode end-plate was made of Pt-Ru black catalyst, assembled with a Nafion™ membrane. For potentiostatic format measurement, a Palmsens mod. EmStat potentiostat (Houten, The Netherlands) was used, connected to the fuel cell, with the PSTrace Software ver. 4.6 data interface, supplied on a Compaq Presario PC. For each measurement, the current was recorded until the steady state at which the supplied current value was read. The fuel cell was connected to Emstat using the anode as the working electrode, while the cathode acted as reference and counter electrode.

Before the current measurements, the Emstat automatically measured the open circuit voltage (OCV) value for a time of about 200 s, and then, the anode potential was set to a value of the optimized applied potential (OAP = OCV−100 mV) [25,26,27].

### 2.2. Chemometric Methods

For the qualitative analysis, principal components analysis (PCA) [29,30,31], which is one of the most well-known chemometric methods commonly used for different purposes [32,33], was performed. The technique is based on the idea that information present in a data matrix **X** can be summarized and compressed in a set of latent variables (linear combination of the original ones) called principal components (PC). From the mathematical point of view, this is achieved by the bilinear decomposition of **X**, which is:

**X** = **TP^T^** + **E** (1)

where **T** is the scores or principal components matrix, **P** is the loadings matrix, which collects the coefficients relating the original variables to the latent ones, and **E** are the residuals, i.e., a matrix containing the variability not explained by the model.

The analysis of a dataset by PCA is conducted by an investigation of the model parameters; for instance, the inspection of the score plots allows for identifying grouping tendencies among the samples. More details on the interpretation of PCA models can be found in [29,30,31,34].

Data was processed by PCA analysis using in-house routines written in MATLAB.

In order to have a better insight into the characteristics of the studied systems, the data were further analyzed by power-slicing followed by Parallel Factor Analysis (PARAFAC) data processing. This approach takes advantage of the holographic nature of the exponential decays and rearranges the data into a three-way structure that can then be processed by appropriate multiway algorithms (in this case, PARAFAC).

**Figure 1:**
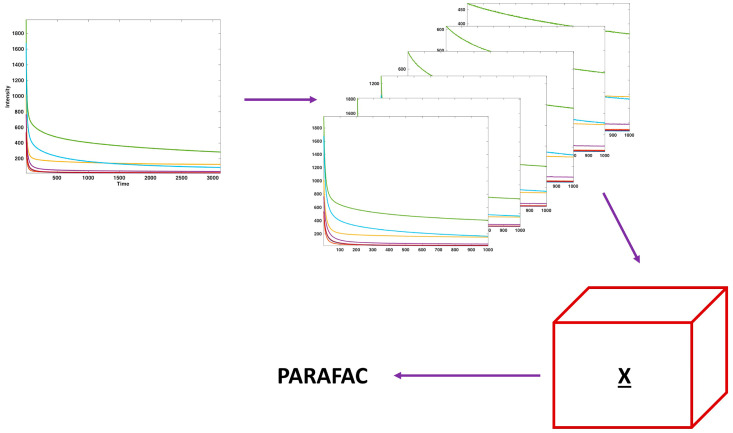
Schematic representation of the slicing procedure. PARAFAC: Parallel Factor Analysis.

Power slicing is a technique proposed in the field of low-resolution NMR in order to transform exponentially decaying profiles into matrices, in order to exploit the advantages of a multiway analysis to process the data. For each sample, to obtain a matrix out of an exponential decay, the profile is shifted a certain number of points (the lag) and added as a second dimension, and this procedure can be repeated a certain number of times, each time changing the entity of the lag, as shown in Figure 1.

In power slicing, as the name suggests, the lags are defined as the power of 2, up to the level the number of data points allows [35]. When the procedure is repeated on more than one exponential profile, i.e., for more than one sample at a time, then the resulting data structure becomes a three-way array, which can be analyzed by means of specifically designed algorithms, such as PARAFAC [36].

PARAFAC is a trilinear decomposition technique that can be considered as the analog of PCA when dealing with multiway data structures. Indeed, the generic element of the data cube **X** (*x*_ijk_) is decomposed as the product of three sets of loadings (one of which can be considered as the scores, if one dimension corresponds to the samples):

(2) $$x_{ijk}=\sum_{f=1}^{F}a_{if}b_{jf}c_{kf}+e_{ijk}$$

where *F* is the number of extracted components; *a_if_*, *b_jf_* and *c_kf_* are the elements of the loading matrices **A**, **B** and **C**, respectively, and *e_ijk_* is the element of the residual cube **E**. Differently than in the case of PCA, PAFARAC components are not orthogonal and, in general, can be directly interpreted as the profiles of the chemical constituents of the analyzed mixtures.

PARAFAC models were built using the PLS-Toolbox (v.8.1, Eigenvector Research Inc., Manson, WA, USA) under a MATLAB environment (The MathWorks, Natick, MA, USA).

Lastly, to take simultaneously into consideration the information from two blocks of data in an unsupervised fashion, common component and specific weight analysis (CCSWA, also known as ComDim [37]), was employed. ComDim was originally proposed for the analysis of sensory data but has found applications in different fields [38,39,40]; it can be considered as a multiblock analog of principal component analysis, in which attention is focused on extracting components that summarize common information among the different blocks of data under investigation. The contribution of the different blocks to the overall description of the component is called the salience and can be inspected for interpretation, i.e., to understand how the different data matrices define the variance of a particular latent variable. In ComDim, a single set of scores is extracted from all the data blocks involved; afterwards, given the scores, loadings are calculated for each matrix, and they can be used for the interpretation of the observed variance.

Briefly, by calling the two blocks of data ***X***_1_ and ***X***_2_, the ComDim algorithm starts by normalizing each block so as to make their variance comparable and building the matrices ***W***_k_ as:

(3) $$W_{1}=X_{1,norm}X_{1,\;norm}^{T}\;W_{2}=X_{2,norm}X_{2,\;norm}^{T}$$

***X***_1,*norm*_ and ***X***_2,*norm*_ being the data matrices after normalization. The main characteristic of ComDim is that the same set of components ***Q*** (in particular, of scores) is used to describe the variability in each of the data blocks:

(4) $$W_{k}=Q\Lambda Q^{T}=\sum_{i=1}^{F}\lambda_{i}^{\left(k\right)}q_{i}q_{i}^{T}$$

where the orthonormal scores of the *F* common components $q_{i}$ are collected in the matrix Q, while the diagonal matrix Λ contains the saliences $\lambda_{i}^{\left(k\right)}$, which represent the variance explained by the *i*-th common component for the *k*-th block.

The three methods adopted are all explorative methods with very limited computation costs, which have different characteristics and, therefore, may provide complementary information on the analyzed data. Indeed, while PCA provides the most parsimonious summary of the variability observed in the current response matrix, analyses of the same decays by slicing and PARAFAC allow to obtain a finer deconvolution of the exponential curves; on the other hand, the multiblock nature of ComDim is the basis to relate the variability observed in the current decays with the parameters of the linear calibrations.

## 3. Results and Discussion

### 3.1. Qualitative Analysis

**Figure 2:**
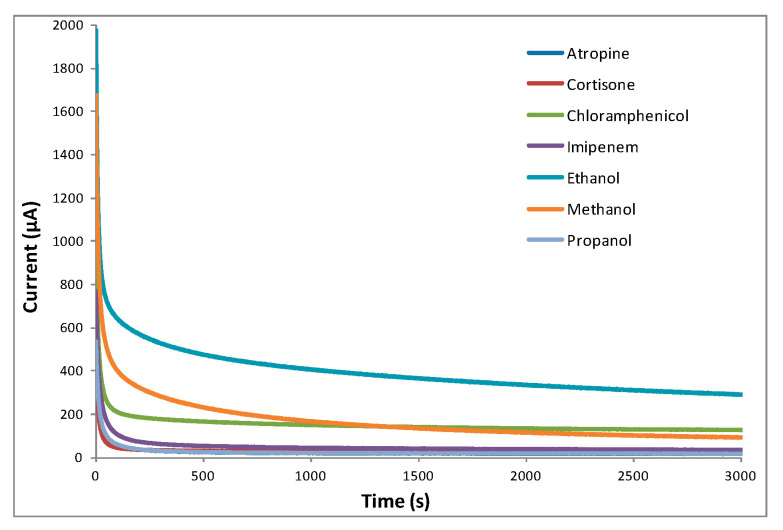
Current response trends of 6 × 10^−4^ mol L^−1^ aqueous solutions of the seven investigated compounds, all data of which constitute the dataset under investigation.

Current response trends to seven investigated organic molecules have been recorded and reported in Figure 2. Each curve is the mean of at least three experiments.

**Figure 3:**
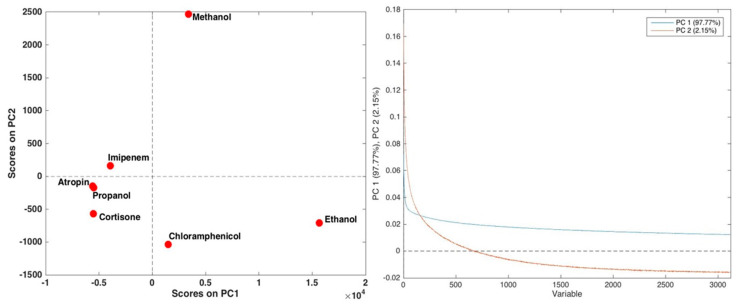
Results of principal component analysis (PCA) on the analyzed dataset: scores plot (**left panel**) and loading plot (**right panel**).

At first, principal component analysis was applied to the dataset obtained by all the data points of each curve response trend after mean centering, and two components (accounting for more than 99.9% of the total variance) appeared to be significant according to cross-validation. The results of PCA are graphically displayed in Figure 3, in terms of scores and loading plots.

Information about the differences in behavior among the analyzed substances can be appreciated by inspecting the scores plot reported in Figure 3. As a whole, the plot shows a relevant difference between ethanol, which is situated at high values of PC1, methanol, which is located at a high level of PC2, and the remaining analytes. Among the latter, chloramphenicol seems to be less similar to the others. In order to try to interpret the observed differences in terms of the response curve behavior, the loadings along the two significant components, reported as well in Figure 3, should be inspected. Loadings along both components indicate that the two components may be ascribable to two response curve trends, with different time constants: in particular, the first component (reported in blue in the plot) should correspond to a process characterized by a higher rate constant, resulting in a faster decay; on the other hand, the second component (in orange) points to a slower process. Accordingly, by comparing the score and the loading plots, one could hypothesize that ethanol and, to a lesser extent, methanol and chloramphenicol are the analytes for which the contribution of the fast-response component is higher, whereas methanol and, to a significantly lesser extent, imipenem present a significant contribution of the slower component.

**Figure 4:**
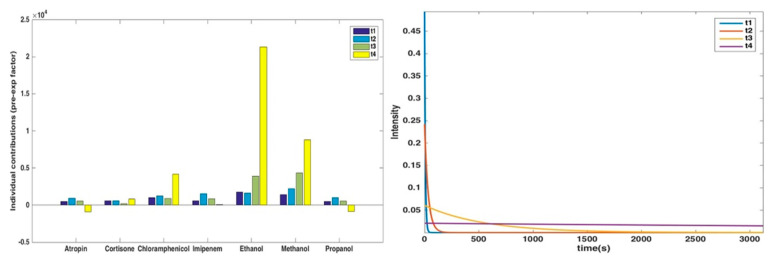
Results of Slicing/PARAFAC on the analyzed dataset: relative contributions of the different exponential response curves (left), whose profiles are reported in the right panel.

The application of PARAFAC [36] to power-sliced exponential data provides, as the outcome, information on the number of response curve components, on their characteristic half-times and on the relative populations of the components across the samples. Accordingly, when the power-slicing approach was applied to the dataset, it was possible to identify four exponential current response trends (with time constants 7.1 s, 33.0 s, 539.0 s and 9502.6 s, respectively) accounting for practically all the variance present in the data and to evaluate the contribution of each of these four exponential trends to the behaviors of the different compounds. The results are reported in Figure 4.

The figure highlights how, in general, the current drop is higher for ethanol, methanol and chloramphenicol, but also that, for these compounds, the slower current components are more relevant. On the other hand, the other compounds show a lower overall intensity drop and, when looking at the individual contributions, a higher amount of the faster current components.

Lastly, in order to simultaneously consider the data from the calibration curves, which show different slope values, for each of the seven organic compounds considered, and from the current response curves already analyzed by PCA and PARAFAC modeling, a multiblock (data fusion) approach based on the ComDim model was carried out. As described in Section 2.2, ComDim extracts components that are common for the data matrices under investigation and, therefore, allow the simultaneous description of the variability of the samples based on all the available experimental data. ComDim was applied on the two blocks of data made of the slopes and intercepts of the calibration curve reported in Table 1 and on the current response curves after autoscaling and mean centering, respectively.

**Figure 5:**
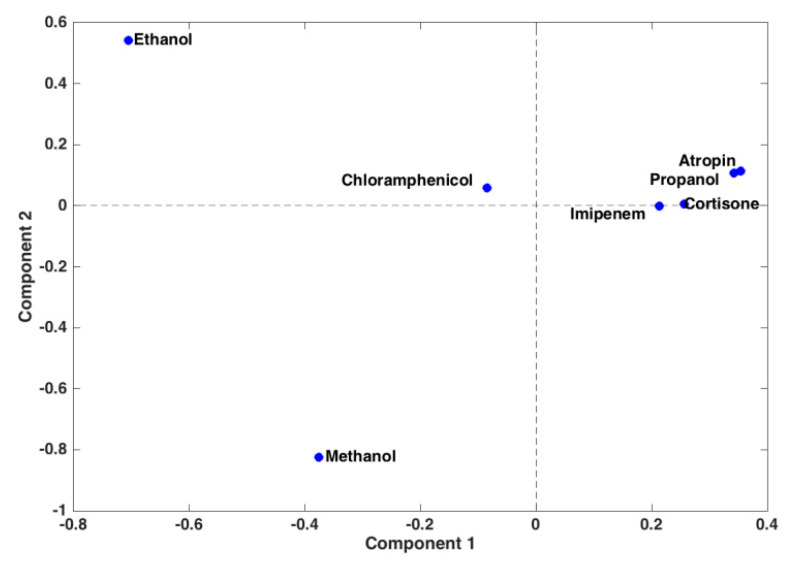
Results of the Common Components and Specific Weight Analysis (ComDim): scores plot.

**Figure 6:**
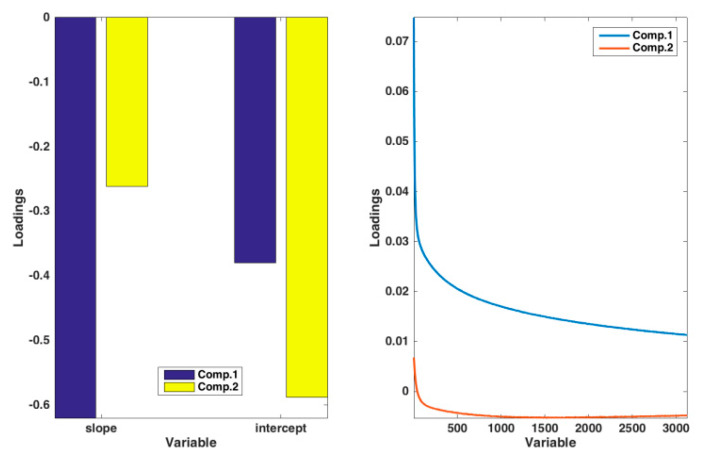
Results of the ComDim: loadings for the two blocks.

The model extracted two significant components, and the projection of the samples onto these common latent variables is reported in Figure 5. Inspecting the figure, one can observe that the trend observed in Figure 3, when analyzing the current response curves only, is confirmed by the multiblock analysis, even if, in Figure 5, atropine and propanol, and imipenem and cortisone, appear more similar to one another. Inspection of the saliences of the two blocks in the description of the latent variables indicates that, both for component 1 and component 2, the two matrices almost contributed equally to the definition of the model subspace. In order to be able to chemically interpret the observed variation among the samples, loadings for the two blocks in the definition of the two components were calculated, and they are displayed in Figure 6. When looking at the contributions from the response curves, as already discussed for Figure 3, two decay trends (one faster and one slower) are observed. In the case of the multiblock analysis, the slower curve is associated to the first component and corresponds to more negative slopes of the calibration curves. On the other hand, the faster decay is associated to the second component and, in particular, to more negative values of the intercept, with a less relevant contribution of the slope term.

### 3.2. Quantitative Analysis

**Figure 7:**
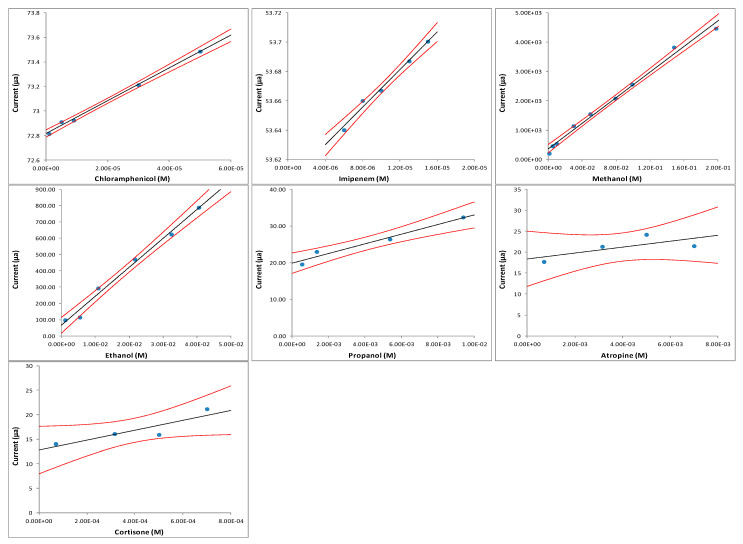
Calibration curves for the seven studied organic analytes.

From a quantitative point of view, of course, the traditional approach can be followed: seven different calibration curves were constructed (one for each studied compounds), each of which allows the quantitative determination of one of the studied organic molecules. The obtained calibration curves are reported in Figure 7. The different calibration curves have been reported in this figure, while their corresponding univariate calibration equations and all analytical data obtained are collected in Table 1; the results indicate significant differences in the sensitivity of the fuel cell to the seven analytes considered.

By using the obtained calibration curves, it is possible to perform a traditional quantitative analysis—however, with different sensitivity and linear ranges for all different considered compounds. As the purpose of this work is not (for the moment) of an application type but, rather, of innovation and methodological development concerning the use of chemometric tools to characterize the electrochemical behavior of different substances (which is what in the paper is also referred to as qualitative analysis, since attention is focused on illustrating the qualitative differences between the analytes’ behaviors), we preferred not to include an excessive amount of experimental details, which, anyway, have been discussed in other papers, and simply summarized the results of the calibrations and the characteristics of the corresponding curves: each of the calibration curves has been reported in the work, and all the analytical data relating to them have been summarized in Table 1. Since the study is a proof of concept to show the potential of this innovative methodological approach, based on the combination of direct methanol fuel cell with advanced chemometric tools, at this stage, concentration ranges that may not be the ones found in some real situations were investigated. Anyway, these analytical data are already completely indicative and coherent for any type of quantitative application to real samples, unless there are possible interferences. On the other hand, as already seen in the previous paragraph (Section 3.1), the different calibration sensitivity values of the different calibration curves have also proved very useful for qualitative analysis, as they have allowed the application of the “ComDim” method, i.e., a multiblock analog of principal components analysis. Concerning the quantitative analysis, the sensitivity, precision, accuracy and limit of detection (LOD) are, of course, different for each compound considered. Looking at Table 1, it can be observed how, for example, the calibration sensitivity varies of about an order of magnitude for the seven substances examined (from about 1000 to about 13,000), while the precision, evaluated as “pooled” (that represents a kind of % SD value along the entire linearity range), varies between 5.7% and 8.5%. However, the latter was found to be only between 0.07% and 0.4%, for example, for the determination of ethanol in alcoholic drinks [26]. On the other hand, as far as the accuracy, estimated by recovery tests (i.e., using the standard addition method), is concerned, it is obvious that it also depends on the compound under examination, given the different sensitivities of the fuel cell towards them; for example, for Imipenem, it was found to be between −3.9% and −4% [28], for Chloramphenicol between −7% and −8% [41], while, for ethanol, from −0.3% to +8% [26]. The LOD value also varies between about 10^−6^ mol L^−1^ and 10^−4^ mol L^−1^, according to the different compounds examined. This means that, generally, there is no difficulty, for example, in analyzing antibiotics tested in pharmaceutical specialties [28,41] or to determine ethanol in wines, beers or hard liquor drinks [25,26]. On the other hand, it is clear that it would be difficult to apply the method to real samples containing concentrations of antibiotics considered to be lower than about 10^−6^ mol L^−1^, or of ethanol, methanol and propanol lower than about 10^−4^ mol L^−1^, without a preliminary preconcentration step. However, it should be noted that, for example, for ethanol or methanol, the LOD depends very much on the catalyst used in the fuel cell. From this point of view, the research, aimed at creating new and more efficient catalysts (also using biocatalysts), is very active, so that it will be probably be possible to significantly lower the current values of the LOD.

## 4. Conclusions

In previous first analytical applications, the DMFC device was used to check the ethanol content in several alcoholic beverages [26]; on the other hand, in the present research, the qualitative and quantitative determination of seven different organic compounds of pharmaceutical or biomedical interest were investigated. For qualitative analysis, the recorded signals of the fuel cell as a function of time (s) for the seven studied organic compounds were processed by chemometrics, and the results obtained from the proposed approach demonstrated that the recognition of each of the seven considered organic compounds (i.e., qualitative analysis), based only on the use of a DMFC-type sensor and chemometric methods, is possible, fast and cheap; in addition, the results are innovative and seem promising in the light of further and wider developments. Lastly, we would like to clarify that, at least for the moment, it is not within our scope to submit the method illustrated in this work to the approval of the EU official legislation. As already mentioned, we consider the present work as research for the development of new analytical possibilities—in particular, concerning the use of chemometric tools to analyze the signal coming from a fuel cell and to characterize the corresponding electrochemical behaviors of the analyzed substances. For quantitative analysis, the classical approach, i.e., by constructing seven different calibration curves (one for each studied compounds), was demonstrated to be suitable: the DMFC device allows the quantitative determination of each one of the considered compounds, with a SD% ≤ 7–8% and a LOD of about 10^−6^ to 10^−4^ mol L^−1^, according to the selected species.

## Figures and Tables

**Table 1:** Summary of the results of univariate calibrations for the various analytes.

	Regression Equation (Y = μA, X = mol L^−1^)	Linearity Range (=mol L^−1^)	R^2^ ^(a)^	Pooled SD	LOD ^(b)^ (=mol L^−1^)
Chloramphenicol	Y = 13.4 × 10^3^ (±5.4 × 10^3^) X + 72.8 (±15.8)	(1.0 × 10^−6^–5.0 × 10^−5^)	0.9961	5.9	9.0 × 10^−7^
Imipenem	Y = 64.0 × 10^2^ (±15.5 × 10^2^) X + 53.6 (±14.1)	(6.0 × 10^−6^–1.5 × 10^−5^)	0.9868	6	5.0 × 10^−6^
Methanol	Y = 21.8 × 10^3^ (±0.78 × 10^3^) X + 0.37 × 10^3^ (±0.07 × 10^3^)	(1.0 × 10^−3^–2.0 × 10^−1^)	0.9912	7.2	8.0 × 10^−4^
Ethanol	Y = 17.8 × 10^3^ (±0.95 × 10^3^) X + 0.07 × 10^3^ (±0.02 × 10^3^)	(1.0 × 10^−3^–4.0 × 10^−2^)	0.9888	6.8	8.0 × 10^−4^
Propanol	Y = 13.2 × 10^2^ (±1.8 × 10^2^) X + 19.8 (±1.0)	(5.4 × 10^−4^–9.4 × 10^−3^)	0.9648	5.7	5.0 × 10^−4^
Atropine	Y = 70.6 × 10^1^ (±49.2 × 10^1^) X + 18.4 (±2.2)	(7.0 × 10^−4^–7.0 × 10^−3^)	0.5076	7	6.5 × 10^−4^
Cortisone	Y = 10.1 × 10^3^ (±3.6 × 10^3^) X + 12.8 (±1.7)	(7.0 × 10^−5^–7.0 × 10^−4^)	0.7956	8.5	6.5 × 10^−5^

(a) R^2^: coefficient of determination; (b) LOD: limit of detection.
